# Optogenetic Globus Pallidus Stimulation Improves Motor Deficits in 6-Hydroxydopamine-Lesioned Mouse Model of Parkinson’s Disease

**DOI:** 10.3390/ijms24097935

**Published:** 2023-04-27

**Authors:** Sonia Di Bisceglie Caballero, Aurelia Ces, Martine Liberge, Frederic Ambroggi, Marianne Amalric, Abdel-Mouttalib Ouagazzal

**Affiliations:** Aix-Marseille Université, CNRS, LNC (UMR 729), 13331 Marseille, France

**Keywords:** external globus pallidus, motor behavior, mice, optogenetic, Parkinson’s disease

## Abstract

Excessive inhibition of the external globus pallidus (GPe) by striatal GABAergic neurons is considered a central mechanism contributing to motor symptoms of Parkinson’s disease (PD). While electrophysiological findings support this view, behavioral studies assessing the beneficial effects of global GPe activations are scarce and the reported results are controversial. We used an optogenetic approach and the standard unilateral 6-hydroxydopamine nigrostriatal dopamine (DA) lesion model of PD to explore the effects of GPe photostimulation on motor deficits in mice. Global optogenetic GPe inhibition was used in normal mice to verify whether it reproduced the typical motor impairment induced by DA lesions. GPe activation improved ipsilateral circling, contralateral forelimb akinesia, locomotor hypoactivity, and bradykinesia in 6-OHDA-lesioned mice at ineffective photostimulation parameters (532 nm, 5 Hz, 3 mW) in normal mice. GPe photoinhibition (450 nm, 12 mW) had no effect on locomotor activity and forelimb use in normal mice. Bilateral photoinhibition (450 nm, 6 mW/side) reduced directed exploration and improved working memory performances indicating that recruitment of GPe in physiological conditions may depend on the behavioral task involved. Collectively, these findings shed new light on the functional role of GPe and suggest that it is a promising target for neuromodulatory restoration of motor deficits in PD.

## 1. Introduction

Basal ganglia (BG) are a highly organized network of subcortical nuclei that controls various aspects of voluntary motor movements and were implicated in many neurodegenerative disorders, including Parkinson’s disease (PD) [1,2]. The external globus pallidus (GPe) was traditionally considered a simple relay nucleus within the network, connecting the striatum and subthalamic nucleus (STN) in the indirect pathway [2,3]. However, recent anatomical and electrophysiological studies uncovered a diversity of GABAergic neurons and anatomical connectivity suggesting that the GPe may serve a much more complex function than just a relay station [4,5,6]. GABAergic neurons of GPe comprise distinct neuronal subpopulations that can be identified based on their electrophysiological properties, molecular features, and projection targets. The two major classes are prototypic neurons that send widespread projections within and outside the BG and arkypallidal neurons that project back to the striatum [4,5,6,7,8]. Despite surge of interest over the past years, our understanding of the functional importance of GPe within the BG circuits remains incomplete. Early behavioral studies showed that pharmacological manipulations with GABAergic and glutamatergic agents that enhance GPe activity promote motor behaviors [9]. Unilateral GPe activations induce contralateral circling in rats and dyskinesia in monkeys while bilateral stimulations produce locomotor hyperactivity in rats [10,11,12,13,14,15,16]. Consistent with pharmacological studies, unilateral optogenetic GPe activation was also reported to induce contralateral circling and hyperkinesia in mice [17]. While these findings consistently show that the net effect of global GPe activation is the facilitation of motor behaviors, studies assessing the impact of cell-type specific optogenetic stimulations revealed a dissociable contribution of GABAergic neuron subtypes to motor behavior [18,19,20,21].

In PD conditions, the degeneration of nigrostriatal dopaminergic neurons triggers a range of functional compensatory changes in BG circuits that lead to the development of motor symptoms [22]. Excessive inhibition of GPe neurons by striatal GABAergic neurons of the indirect pathway is considered a critical mechanism underlying the pathological overactivity of the subthalamic nucleus and BG output structures and thus the expression of the motor symptoms [1,6,22,23]. While electrophysiological and neurochemical studies in patients and animal models of PD provide support for this view [24,25,26,27,28], few studies have explored the behavioral effects of global GPe stimulations in the context of dopamine (DA) depletion, and the reported results are controversial. Pharmacological and global chemogenetic GPe activations ameliorate motor deficits in reserpine-treated rats and in mice with bilateral medial forebrain bundle (MFB) lesions, respectively [29,30,31]. By contrast, no effect was found with either global optogenetic GPe activation or inhibition in MFB-lesioned mice [32]. Whether the discrepancy is due to the difference in neuromodulation approaches used is still an open question. Addressing this issue is important from a therapeutic standpoint as optogenetic technology has entered clinical trials for retinal degenerative diseases and has the potential to expand into brain disorders [33,34], such as PD for which deep brain stimulation (DBS) therapy is well-established.

In the present study, we used the standard unilateral 6-hydroxydopamine (6-OHDA) nigrostriatal dopamine (DA) lesion model in mice to reevaluate the potential therapeutic effects of global optogenetic GPe stimulation on motor deficits. The behavioral effects of optogenetic inhibition of GPe were also studied in normal mice under the same testing conditions to examine whether the reduction of GPe activity reproduces the typical motor deficits of DA lesions. To date, it remains also unclear whether GPe inhibition in physiological conditions produces motor deficits like DA lesions. While pharmacological inhibitions of GPe produce motor impairments in rats [10,11,35,36], recent optogenetic studies targeting striatal indirect pathway and GPe found no effects in mice [37,38], thus raising a question about GPe participation in locomotor behavior in physiological conditions. The findings show that global GPe photostimulation improves a range of motor deficits in 6-OHDA-lesioned mice. In normal mice, GPe photoinhibition had little effect on motor behavior but promoted cognitive functions. These findings shed new light on GPe function and show that this BG nucleus differentially contributes to the modulation of motor functions in normal and PD conditions.

## 2. Results

### 2.1. GPe Photostimulation Improves Ipsilateral Circling Behavior and Contralateral Forelimb Akinesia in 6-OHDA-Lesioned Mice

**Figure 1:**
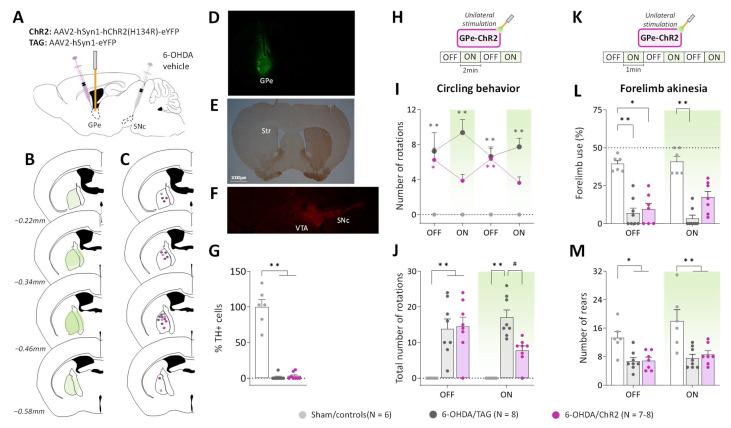
GPe photostimulation ameliorates circling behavior and forelimb akinesia in 6-OHDA-lesioned mice. (**A**) Schematic illustration of the experimental procedure for opsin expression in GPe and nigrostriatal DA lesion. (**B**) Illustration of the immunofluorescence spreading across the anteroposterior axis in the brain sections of 6-OHDA/ChR2 mice containing the GPe. (**C**) Placement of individual optical fiber across the anteroposterior axis of the GPe for all experimental groups. (**D**) Immunofluorescence image illustrating unilateral eYFP expression in GPe neurons. (**E**) DAB immunostaining for TH-positive fibers in the striatum of a lesioned mouse. (**F**) Immunostaining for TH+ cells in SNc of a lesioned mouse. (**G**) Percentage of TH+ cell loss in SNc in lesioned mice relative to Sham mice. (**H**) Photostimulation protocol used for the open field test. Testing was carried over 8 min with repeated sequences of light pulses stimulation (532 nm, 5 Hz, 5 ms, 3 mW) turned OFF and ON for 2 min. (**I,J**) Time course and a total number of spontaneous ipsilateral rotations during OFF and ON periods, respectively. (**K**) Photostimulation protocol was used for the cylinder test. Testing was carried over 6 min with repeated sequences of light pulses stimulation (532 nm, 5 Hz, 5 ms, 3 mW) turned ON and OFF for 1 min. (**L**) Percentage (%) of contralateral forelimb use during OFF and ON periods (number of contralateral forepaw contacts relative to the total number of forepaw contacts). (**M**) Total number of rears (forepaw contacts) during OFF and ON periods. Sham/controls, N = 6; 6-OHDA/TAG, N = 8; 6-OHDA/ChR2, N = 7–8. One mouse from the 6-OHDA/ChR2 group was excluded from the cylinder test (see Methods). Results are presented as mean ± SEM. * *p* < 0.05 and ** *p* < 0.01 significantly different from Sham mice. # *p* < 0.05 significantly different from 6-OHDA/TAG mice, Dunn’s test following a significant non-parametric Kruskal–Wallis test.

To examine whether GPe activation improves motor impairments induced by unilateral DA lesion, the excitatory channelrhodopsin 2 (ChR2) variant, ChR2(H134R), which has a larger photocurrent than wildtype ChR2 [39], was expressed in GPe neurons under the control of the human synapsin-1 (hSyn1) promoter using an adeno-associated virus (AAV) vector (Figure 1A). Only mice with successful opsin expression (Figure 1B,D) and optic fiber implantation (Figure 1C) were included in the study. Immunohistochemical staining of tyrosine hydroxylase (TH) confirmed the complete loss of dopaminergic fibers in the striatum of lesioned mice (Figure 1D), and quantitative analysis of TH immunofluorescence in the substantia nigra pars compacta (SNc) revealed over 98% loss of TH positive cells in 6-OHDA/TAG and 6-OHDA/ChR2 mice compared to Sham/controls (*p* ≤ 0.005 for both groups, Dunn’s post hoc test following a significant Kruskal–Wallis test, Figure 1E,F).

To reveal potential alterations of GPe activity, pilot studies were conducted in a viewing jar (see Methods) in order to select the optimal optical stimulation parameters that are behaviorally ineffective in normal mice. Consistent with previous studies (see introduction), unilateral GPe photostimulation (532 nm, 40–10 Hz frequencies) produced locomotor asymmetry in mice with turning contralateral to the illumination side (Supplementary Figure S1C). The number of contralateral circling decreased as a function of the light pulse parameters (*p* < 0.05 vs. OFF period for 40 and 30 Hz, Mann–Whitney test, Supplementary Figure S1C) reflecting reduced GPe activation. A lower illumination frequency (5 Hz, 5 ms, 3 mW) was then used for studying the impact of GPe activation on ipsilateral circling and forelimb akinesia induced by the unilateral 6-OHDA lesion. The 5 Hz photostimulation triggered no contralateral circling (null scores, Figure 1H–J) and produced no change in spontaneous locomotor activity and forelimb use in non-lesioned control mice expressing the opsin (Sham/ChR2) or the fluorescent protein alone (Sham/TAG, Supplementary Figure S1). Though, a motor stimulant effect was still apparent in rearing behavior in the cylinder test (*p* < 0.05, Mann–Whitney test, Supplementary Figure S1I). The two control groups, Sham/ChR2 and Sham/TAG mice were pooled together in this study (Sham/controls, Figure 1). During the pre-stimulation period (first OFF, Figure 1H), 6-OHDA/TAG and 6-OHDA/ChR2 mice had comparable ipsilateral circling scores that were significantly higher than those of Sham/controls (*p* ≤ 0.02, non-parametric Dunn’s post hoc test). The photostimulation had no effect in 6-OHDA/TAG mice (*p* < 0.0005 for all ON periods vs. Sham/controls, Dunn’s post hoc test), but it reduced ipsilateral circling in 6-OHDA/ChR2 mice (*p* > 0.05 vs. Sham/controls, Dunn’s post hoc test, Figure 1H). The improvement immediately vanished when the optical stimulation was turned OFF. Analysis of cumulative circling scores over the OFF and ON periods revealed a significant main effect of the lesion (H(3) ≤ 16.58 for both conditions, *p* < 0.0005, Kruskal–Wallis test; Figure 1I) and post hoc test confirmed a beneficial effect of the photostimulation only in 6-OHDA/ChR2 mice (*p* > 0.05 vs. Sham/controls, Dunn’s post hoc test).

We next verified whether GPe activation could restore contralateral forelimb akinesia in the cylinder test. Forelimb use in the cylinder test is associated with postural support when the mouse is up on its hind limbs and is a reliable index of akinesia induced by dopaminergic depletion. Figure 1K illustrates the global score of contralateral forelimb use in the OFF and ON periods. A significant impairment was detected in all lesioned groups during the OFF periods (*p* ≤ 0.01 vs. Sham/controls, Dunn’s post hoc test). Application of the optical stimulation had no effect in 6-OHDA/TAG mice (*p* < 0.001, Dunn’s post hoc test), but improved contralateral forelimb use deficit in 6-OHDA/ChR2 mice (*p* > 0.05, Dunn’s post hoc test). The total number of rearing was significantly reduced in both lesioned groups (main effect of 6-OHDA lesion, F_(2, 18)_ = 11.18, *p* < 0.001, Figure 1L), and GPe photostimulation was ineffective (*p* < 0.05 for both lesioned groups vs. Sham/controls, Tukey’s post hoc test, Figure 1L).

### 2.2. GPe Photostimulation Improves Locomotor Hypoactivity and Bradykinesia in 6-OHDA-Lesioned Mice

**Figure 2:**
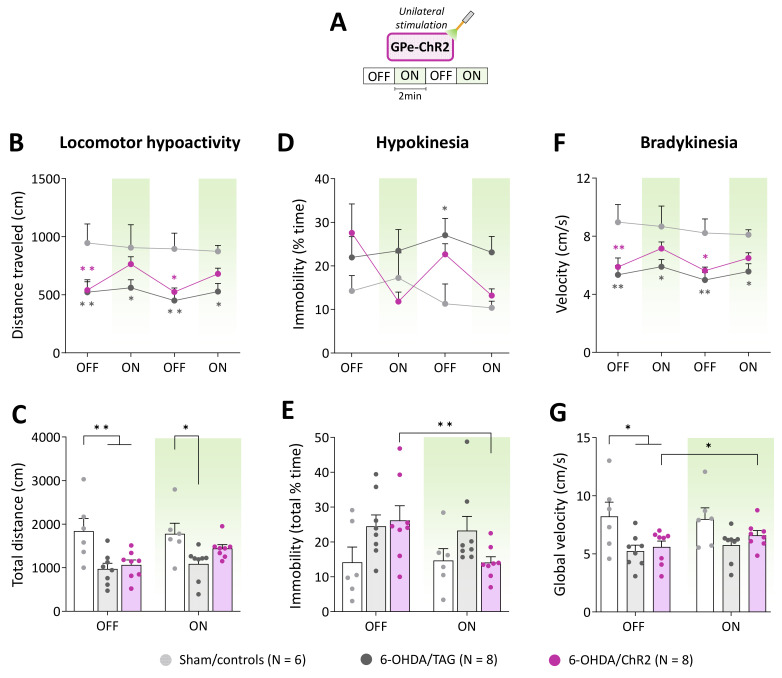
GPe photostimulation ameliorates locomotor activity measures in 6-OHDA-lesioned mice. (**A**) Photostimulation protocol used for the open field test. Testing was carried over 8 min with repeated sequences of light pulses stimulation (532 nm, 5 Hz, 5 ms, 3 mW) turned OFF and ON for 2 min. (**B**,**C**) Time course and total distance traveled (cm) during OFF and ON periods, respectively. (**D**,**E**) Time course and total time spent in immobility (%) during OFF and ON periods, respectively. (**F**,**G**) Time course and global locomotor velocity during OFF and ON periods, respectively. Sham/controls, N = 6; 6-OHDA/TAG, N = 8; 6-OHDA/ChR2, N = 8. Results are presented as mean ± SEM. * *p* < 0.05 and ** *p* < 0.01 significantly different from Sham mice or within group, Tukey’s test following a significant two-way repeated measures ANOVA.

To further characterize the beneficial effects of GPe activation in lesioned mice we analyzed various locomotor activity parameters. Figure 2B illustrates the time course of the distance traveled in the open field arena upon repeated application of the photostimulation (532 nm, 5 Hz, 5 ms, 3 mW, Figure 2A). In the pre-stimulation period, 6-OHDA/TAG and 6-OHDA/ChR2 mice had significantly lower baseline locomotor activity levels than Sham/controls (*p* < 0.01 for both groups, Tukey’s post hoc test). GPe photostimulation increased locomotor activity in 6-OHDA/ChR2 mice (*p* > 0.05 vs. Sham/controls, Tukey’s post hoc test) but not in 6-OHDA/TAG mice (*p* < 0.05 vs. Sham/controls, Tukey’s post hoc test). Repeated measures ANOVA performed on global scores (Figure 2C) yielded a significant main effect of 6-OHDA lesion (F_(2, 19)_ = 6.12, *p* < 0.05) and optical stimulation (F_(1, 19)_ = 7.16, *p* < 0.01). Post hoc analysis confirmed that GPe photostimulation had a beneficial effect only in 6-OHDA/ChR2 mice (*p* > 0.05 vs. Sham/controls, Tukey’s post hoc test).

Hypokinesia assessed by the percentage of time spent in immobility (Figure 2D) was overall higher in lesioned groups compared to Sham/controls (F_(2, 19)_ =2.97, *p* < 0.05, 6-OHDA lesion effect). Though, a significant difference between 6-OHDA/TAG mice and Sham controls was only detected at the 2nd OFF period (*p* < 0.05, Tukey’s post hoc test). The photostimulation reduced % of the time spent in immobility in 6-OHDA/ChR2 in a reversible manner (F_(6, 57)_ = 2.44, *p* < 0.05, 6-OHDA lesion x photostimulation interaction, Figure 2D). Analysis of the global scores (Figure 2E) yielded a significant main effect of optical stimulation (F_(1, 19)_ = 5.06, *p* < 0.05) and a significant 6-OHDA lesion × optical stimulation interaction (F_(2, 19)_ = 4.54, *p* < 0.05, Figure 2E), but the post hoc test failed to reveal a significant difference between groups (*p* > 0.05, Tukey’s test). Further analysis performed within groups confirmed that only 6-OHDA/ChR2 mice had significantly lower scores in the ON than the OFF period (*p* < 0.05, Student’s *t*-test, Figure 2E).

Lesioned mice also displayed a marked bradykinesia illustrated by slower locomotion compared to Sham controls (F_(2, 19)_ = 6.97, *p* < 0.01, 6-OHDA lesion effect, Figure 2F). The average velocity in the OFF periods was around 9 cm/s for Sham/controls and below 6 cm/s for lesioned mice (*p* ≤ 0.04, Tukey’s post hoc test). The photostimulation enhances locomotor velocity in 6-OHDA/ChR2 (F_(3, 57)_ = 2.82, *p* < 0.05) at all ON periods (*p* > 0.05 vs. Sham/controls, Tukey’s post hoc test, Figure 2F). Analysis of the global scores (Figure 2G) revealed a significant main effect of the 6-OHDA lesion (F_(2, 19_)= 3.99, *p* < 0.05) and optical stimulation (F_(1, 19)_ = 5.14, *p* < 0.04) but post hoc test failed to reveal a difference between groups at ON period (*p* > 0.05, Tukey’s post hoc test, Figure 2G). Further analysis performed within groups confirmed that only 6-OHDA/ChR2 mice had significantly higher velocity scores in the ON than the OFF period (*p* < 0.05, Student’s *t*-test, Figure 2G).

### 2.3. GPe Photoinhibition in Normal Mice Does Not Mimic Motor Deficits of DA Depletion

**Figure 3:**
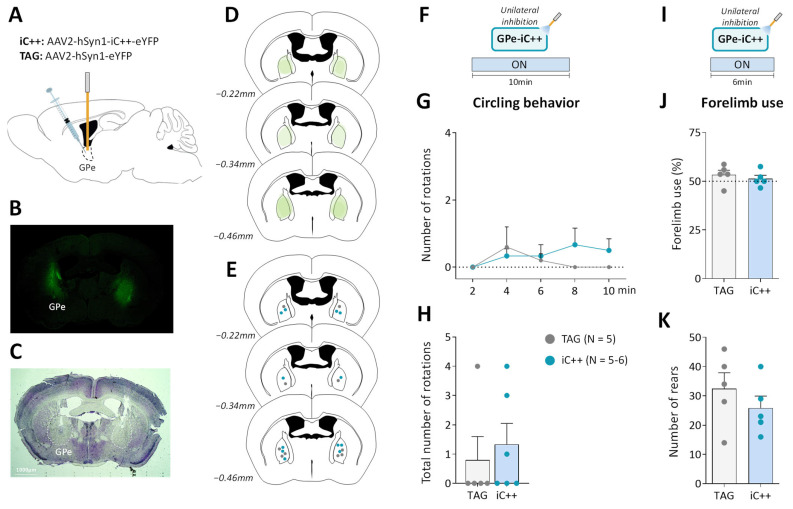
Unilateral GPe photoinhibition does not produce motor deficits. (**A**) Schematic illustration of the experimental procedure for opsin expression in GPe. (**B**) Immunofluorescence image illustrating bilateral viral expression in GPe neurons. (**C**) Cresyl violet staining showing bilateral fiber optic placement in the GPe. (**D**) Illustration of the immunofluorescence spreading across the anteroposterior axis in the brain sections of iC++ mice containing the GPe. (**E**) Placement of individual optical fiber across anteroposterior GPe axis for TAG and iC++ mice. (**F**) Photoinhibition protocol used in the open field test. Testing was carried out over 10 min with continuous light illumination (450 nm, 12 mW). (**G**,**H**) Time course and the total number of ipsilateral rotations over 10 min of testing, respectively. (**I**) Photoinhibition protocol used in the cylinder test. Testing was carried out over 6 min with continuous light illumination (450 nm, 12 mW). (**J**) Percentage of contralateral forelimb use relative to the total number of forepaw contacts. (**K**) Total number of rears (forepaw contacts). TAG, N = 5; iC++, N = 6. One mouse from the iC++ group was excluded from the cylinder test (see Methods). Results are presented as mean ± SEM.

We next examined whether optogenetic inhibition of GPe in normal mice could reproduce the typical motor deficits of the unilateral 6-OHDA lesion. To do so, we used the engineered blue-sensitive chloride-conducting channelrhodopsin, iC++, which allows more efficient optogenetic control than the hyperpolarization induced by light-activated chloride pumps [40]. iC++ was expressed in GPe under the control of the hSyn1 promoter (Figure 3A). Only mice with successful opsin expression (Figure 3B,D) and optic fiber implantation within the GPe (Figure 3C,E) were included in the study. We first examined whether unilateral GPe photoinhibition induces ipsilateral circling. Based on previously published studies [40,41], various illumination parameters (450 nm, frequencies: 30–60 Hz, durations: 5–10 ms duration and intensities: 5–12 mW) were tested using a repeated sequence of light pulses turned OFF and ON in the viewing jar. Only a minor tendency to circle toward the illumination side was detected with a frequency range ≥ 40 Hz. Supplementary Figure S2B,C illustrate, respectively time-course of ipsilateral circling and cumulative circling scores obtained with the highest light pulse parameters tested in the open field arena (60 Hz, 10 ms, 12 mW, *p* > 0.05 for both conditions, Mann–Whitney test). There was also no change in spontaneous locomotor activity assessed by distance traveled (*p* > 0.05, repeated measures ANOVA, Supplementary Figure S2D,E). Continuous photoinhibition [40] with the highest illumination intensity (12 mW) for 10 min produced no notable increase in ipsilateral circling in iC++ mice (*p* > 0.05, Mann–Whitney test, Figure 3G,H). Analysis of locomotor activity measures also did not reveal any changes in distance traveled (*p* > 0.05, repeated measures ANOVA, Supplementary Figure S2G,H), % of the time spent in immobility (*p* > 0.05, repeated measures ANOVA), and velocity (*p* > 0.05, repeated measures ANOVA). In the cylinder test, the unilateral photoinhibition (12 mW, Figure 3I) produced no impairment in contralateral forelimb use (*p* > 0.05, Student’s *t*-test, Figure 3J) and rearing behavior (*p* > 0.05, Student’s *t*-test, Figure 3K).

### 2.4. GPe Photoinhibtion Improves Cognitive Functions in Normal Mice

**Figure 4:**
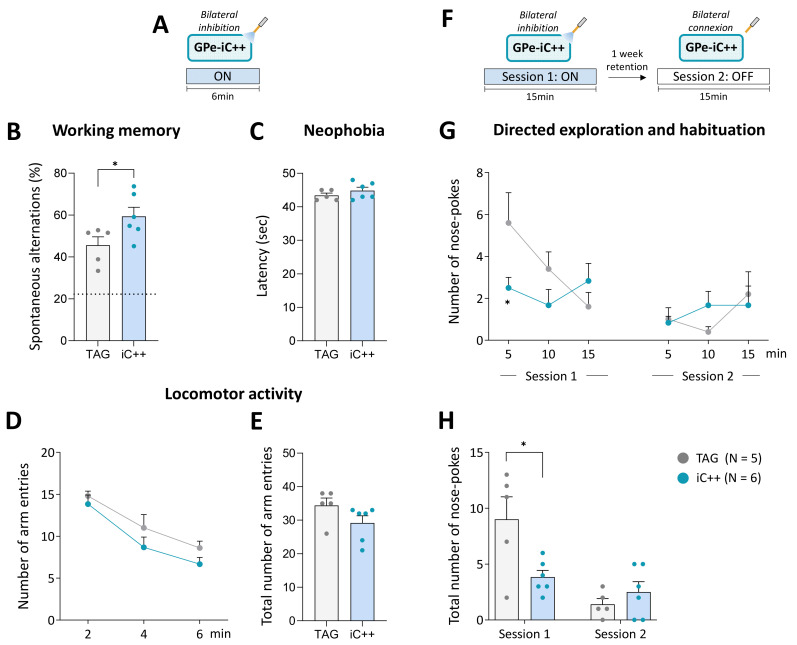
Bilateral GPe photoinhibition impacts working memory and directed exploration. (**A**) Photoinhibition protocol used in the Y-maze test. Testing was carried out over 6 min with continuous light stimulation (450 nm, 6 mW/side). (**B**) Percentage of spontaneous alternations. The dashed line shows a chance level of 22.2%. (**C**) Latency to exit the starting arm (sec). (**D**,**E**) Time course and the total number of arm entries in the Y-maze over 6 min of testing, respectively. (**F**) Photoinhibition protocol used in the nose-poke habituation test. Two 15 min of testing were carried out one week apart, session 1 with continuous light illumination (450 nm, 6 mW/side) and session 2 with light OFF. (**G**,**H**) Time course and total number of nose-pokes per session. TAG, N = 5; iC++, N = 6. Results are presented as mean ± SEM. * *p* < 0.05, unpaired *t*-test for Y-maze test and unpaired *t*-test following a significant repeated measures ANOVA for nose-poke exploration.

The above findings indicate that GPe may not contribute to the modulation of spontaneous locomotor behavior in physiological conditions. GPe establishes direct anatomical connections with the cortex, thalamus, and amygdala [19,42,43,44,45] suggesting that it may participate in the modulation of motivational and cognitive aspects of motor behavior. To explore this possibility, we examined whether bilateral GPe photoinhibition (6 mW/side) could impact short-term spatial working memory performance assessed by spontaneous alternations in the Y-maze task (Figure 4A,B). The latency to exit the starting arm and the number of arm entries were used as an index of neophobia and general locomotor activity, respectively. TAG and iC++ mice, displayed good spontaneous alternation performances that were above chance level (calculated at 22.2%, *p* < 0.05, One-group test, Figure 4B). Interestingly, iC++ mice had a better score than TAG mice (*p* < 0.05, Student’s *t*-test) indicating that the photoinhibition promotes short-term working memory. The photoinhibition did not change the latency to exit the starting arm (*p* < 0.05 vs. TAG mice, Student’s *t*-test, Figure 4C) or the number of arm entries (*p* < 0.05 vs. TAG mice, Student’s *t*-test, Figure 4D,E).

The effect of the photoinhibition was also studied on the long-term habituation to spatial cues, a simple form of non-associative learning, using one week retention delay. Testing was conducted over 15 min in operant chambers equipped with illuminated nose-poke ports placed on the front wall. We previously demonstrated that nose-poke ports can be employed as spatial cues for evaluating directed exploration and learning performances in mice [46,47,48]. Mice received bilateral optical inhibition (6 mW/side) solely on the first session (Figure 4F). TAG mice displayed the expected exploration profile (Figure 4G). In session 1, they initially displayed a high number of nose-poking that declined over the course of the session and then remained at the same low level on the second testing session, reflecting, respectively short-term (within-session) and long-term (between-session) habituation phenomenon (F_(9, 81)_ = 5.062, *p* < 0.0001, main effect across days). iC++ mice had a remarkably low exploration level on session 1 compared to TAG mice (F_(9, 81)_ = 2.574, *p* < 0.05, sessions x optical inhibition interaction), and a significant difference between groups was detected in the first block (*p* < 0.05, Student’s *t*-test). In session 2, they displayed a normal habituation performance illustrated by a low exploration level comparable to TAG mice (*p* > 0.05, Student’s *t*-test for all blocks). Analysis of the global scores confirmed that iC++ mice had a significantly lower number of nose-poking than TAG mice only in session 1 (*p* < 0.05, Student’s *t*-test, Figure 4H) indicating that they were able to efficiently process and retain novel spatial information despite their low exploration level.

## 3. Discussion

The present study shows that global GPe optogenetic activation is effective at reducing motor impairments in 6-OHDA-lesioned mice. As expected, unilateral SNc lesion produced the typical motor deficits in mice manifested by ipsilateral circling, locomotor hypoactivity, bradykinesia, and forelimb akinesia. Global GPe optogenetic activation improved the various motor abnormalities. The beneficial effects were reversible and only seen in 6-OHDA-lesioned mice expressing the excitatory opsin. Mice with optic fiber placement outside of the GPe show no behavioral changes upon application of the illumination ruling out a non-specific effect of the optogenetic manipulation. The improvement of locomotor hypoactivity may be due to the restoration of ipsilateral circling, and thus the balance in the motor circuit, which is essential to walk in a straight line and to travel over long distances in the arena. It may also be attributed to the restoration of the exploration drive as dopaminergic lesions are well known to reduce novelty-induced exploration. The amelioration of hypokinesia and bradykinesia produced by GPe photostimulation is consistent with this conclusion. The photostimulation was also efficient at improving akinesia assessed by contralateral forelimb use in the cylinder test. Overall, our findings extend those reported in the literature using pharmacological and chemogenetic approaches [29,30,31]. However, they contrast with those of Mastro et al. [32] showing that cell-specific rather than global GPe optogenetic manipulations are effective in restoring motor deficits in MFB-lesioned mice, a PD model of severe DA deficiency. The discrepancy between the two studies may be explained by the differences in photostimulation protocols and PD mouse models used. The unilateral intranigral lesion model we used produces a less severe DA depletion and motor impairments than the bilateral MFB lesion that targets all midbrain DA neurons. It is therefore possible that global GPe photostimulation may more readily alleviate motor impairments induced by unilateral SNc than bilateral MFB lesions. It should be stressed that in the Mastro et al. study, the behavioral effects of GPe photostimulation were assessed 3–5 days post-lesion while in our study they were evaluated at a much later time point (5–6 weeks post-lesion). Such a difference in post-lesion testing time may be another possible source of the discrepant results considering that BG circuity undergoes major functional changes several weeks after DA depletion [3]. An interesting study will be to assess the beneficial effects of global GPe photostimulations in 6-OHDA-lesioned mice at different post-lesion time points.

Numerous electrophysiological studies demonstrated a reduction of GPe neuronal firings in animal models and PD patients [3,24,26,28]. GPe hypoactivity has been attributed to an abnormal elevation of extracellular GABA concentrations caused by the overactivity of the striato-pallidal pathway [23,25,49] and to local changes at the level of pallidal GABAergic synapses [27,50]. Our study provides new behavioral evidence that GPe activity is altered upon DA depletion. We show that an ineffective optical stimulation in normal mice is sufficient for improving many aspects of motor impairments caused by DA lesion. Here, we selected low optical stimulation parameters to reveal possible functional alterations of the GPe. It remains possible that the beneficial effects produced by the low illumination frequency (5 Hz) may be attributed to recruitment of specific neuronal population rather than the activation of all GPe neurons. A higher photostimulation frequency (10 Hz, 10 ms, 5 mW) tested in a few animals in the viewing jar completely reversed ipsilateral circling, indicating that increasing the optical photostimulation parameters produces more robust beneficial effects. Though, it produced circling in the opposite direction in Sham/ChR2 mice. Further studies involving electrophysiological recording should help clarify the impact of the various optical stimulation parameters on GPe neuronal activity and select the optimal illumination parameters.

The greater sensitivity of 6-OHDA-lesioned mice to behavioral effects of the photostimulation may reflect a functional hypersensitivity of GPe GABAergic neurons that develops following DA lesion. For instance, the number of GABAergic synapses and the expression of GABA_A_ receptors are increased in the STN upon DA depletion leading to a strengthening of the functional connectivity between GPe and STN [51,52,53]. Hence, the motor improvement seen in lesioned mice may be attributed to the restoration of the inhibitory drive of GPe prototypic neurons expressing parvalbumin (PV-neurons), which are the main source of GPe inputs to STN and BG output structures (substantia nigra pars reticulata and entopeduncular nucleus homologous to primates globus pallidus interna) [4]. Selective optogenetic stimulations of PV neurons were consistently shown to alleviate motor deficits in mouse models of PD [32,54,55]. Interestingly, we found that a behaviorally ineffective optical stimulation in normal mice restores the motor deficits in lesioned mice to normal levels [55]. It is worth noting that not all GPe neurons promote motor behaviors. Optogenetic silencing of prototypic neurons expressing Lim homeobox 6 (Lhx6-neurons) improves motor deficits in MFB-lesioned mice indicating that this neuronal subpopulation plays a deleterious role in the context of DA depletion [32]. Optical stimulations of arkypallidal neurons, namely FoxP2-expressing neurons that exclusively innervate the striatum, were also found to suppress locomotion in normal mice [21,56]. The fact that global GPe photostimulation produces a beneficial effect in 6-OHDA-lesioned mice implies that the recruitment of PV-neurons overrides deleterious influences of other GPe neuronal populations that suppress motor behavior, an action that may be exerted through a local collateral inhibition and suppression of downstream BG nuclei as recently demonstrated [21].

The series of experiments conducted in normal mice show that the mere reduction of GPe activity did not produce impairment in spontaneous locomotor behavior and forelimb use, as DA lesions did. It is unlikely that the illumination parameters used are insufficient for silencing GPe neurons. We tested a range of photoinhibition protocols that were previously demonstrated to be effective for silencing neurons and triggering behavioral responses in normal mice [41,57]. Our results are in agreement with a recent study by Isett et al. [38] demonstrating a small effect of optogenetic inhibitions of GPe and striatal indirect pathway on locomotor activity in mice. They also extend those reported in rats and monkeys showing that neurotoxic lesions of GPe do not reproduce changes in BG discharge patterns and motor dysfunction observed in DA-depleted states [28,58]. Collectively, these findings suggest that GPe contribution to gross motor function may not be critical in physiological conditions. Though, in a Parkinsonian patient, a lesion confined to the GPe exacerbates akinesia [59]. GPe ablation was also reported to worsen motor deficits in Parkinsonian monkeys and to abolish the improvement induced by the dopaminergic receptor agonist, apomorphine [60]. It thus appears that in DA-depleted state, GPe may play a more important role in the development of motor deficits. In PD, the degeneration of DA neurons also leads to a hypoactivity of the direct striatal pathway that promotes motor behaviors. It is therefore possible that the deleterious impact of reduced GPe activity on motor function may be exacerbated when direct striatal pathway transmission is concomitantly reduced. Another possibility could be that the inhibitory drive of some GPe GABAergic neurons that suppress motor behavior is enhanced upon DA depletion. As mentioned above, optical inhibition of GPe Lhx6-neurons improves motor deficits in MFB-lesioned mice [32]. Further, striatal inputs of Npas1^+^-expressing neurons, which suppress locomotor behavior, have been shown to be strengthened upon DA lesion [56]. Finally, it should be noted that motor symptoms of PD were linked not only to changes in local firing rates but also to abnormal rhythmic activity in BG and cortex manifested by enhanced oscillations in the beta frequency range (13–30 Hz) [61,62]. Pathological beta-oscillations are thought to compromise information flow and processing in the cortico-basal ganglia (CBC) network, thereby leading to the impairment of motor functions. Recent preclinical studies showed that GPe plays a central role in this context by promoting the expression and propagation of abnormal beta-oscillations throughout the CBG network [63,64,65,66,67]. Such capability of orchestrating changes in the pattern of activity across the CBG network may thus emerge as a consequence of the compensatory alterations of GPe inhibitory drive onto BG nuclei and thalamus following DA neuron loss [19,51,64]. The contrasting effects of optogenetic GPe modulations on motor functions in normal and 6-OHDA-lesioned mice align with this conclusion.

An important finding was the improvement of cognitive performances produced by GPe inactivation in normal mice. In the Y-maze task, the photoinhibition induced a specific improvement of short-term working memory that was manifested by an increase of spontaneous alternations without any changes in locomotor exploration and emotionality. In the nose-poke habituation task, the promnesic effect was less clear-cut because the photoinhibition reduced nose-poking behavior. Despite the exploration deficit, iC++ mice displayed a normal long-term habituation performance like control mice, which also points to cognitive facilitation. The reduction of nose-poking behavior induced by the photoinhibition contrasts with the null effects found in the open field and the Y-maze tests. In both cases, higher illumination intensities (8–12 vs. 6 mW) produced no locomotor dysfunction in mice, supporting the view that directed exploration and locomotor activity are subserved by distinct neural substrates [46,47,48]. It should be mentioned that selective bilateral chemogenetic inhibition of PV-neurons was recently shown to impair locomotor activity in normal mice [19]. It is therefore possible that optogenetic inhibition targeting a specific GPe neuronal population may be more effective than global manipulations because of the functional heterogeneity of GPe neurons (see also [38]). The low level of nose-poke exploration displayed by iC++ mice in session 1 may also indicate a promnesic action of photoinhibition. An enhanced ability to process and retain spatial information should favor a rapid familiarization of mice with surrounding salient spatial cues and thus lead to reduced investigatory activity. Such cognitive facilitation can explain the good working memory performance of iC++ mice revealed in the Y-maze task, which also requires spatial processing. The precise mechanisms underlying the inhibitory influence of GPe on cognitive processing remains to be delineated. GPe contains a diversity of GABAergic neurons that sends widespread projections within and outside BG and could each influence specific aspects of motor and cognitive behaviors. In support of this idea, it was recently shown that PV neurons projecting to the SNr promote motor behavior while those innervating the parafascicular thalamus negatively modulate behavioral flexibility in mice [19]. It is therefore possible that the promnesic effects produced by the photoinhibition may partly be attributed to the inhibition of GPe inputs to the parafascicular thalamus. It may also be linked to a suppression of the GPe inhibitory drive onto the frontal cortex [42] that subserves executive functions. Future studies are necessary to elucidate the specific contribution of the GPe pathways to the different facets of cognitive behaviors.

In conclusion, our findings provide new insight into the functional role of GPe and its contribution to motor deficits of PD. They show that global GPe photostimulation is effective in improving a range of motor deficits in a 6-OHDA-lesioned mouse model of PD. Reduced GPe activity is considered a key mechanism underlying the pathological overactivity of the STN and thus to the development of PD motor symptoms. Currently, enhancing GABAergic tone in the STN with a viral vector gene transfer technology is explored in the clinic as a therapeutic approach for treating PD [68] (e.g., NCT01301573, NCT05603312). Optogenetic therapy has also entered clinical trials for vision restoration and has the potential to expand into brain disorders [33,34], such as PD for which deep brain stimulation (DBS) is well established. The optogenetic approach will provide an unprecedented ability to precisely control GPe neuronal activity, unlike electrical stimulation that simultaneously targets cells, local afferents, and fibers of passage. Our findings indicate that GPe is a promising target for implementing light-based DBS therapy for PD. They suggest that optical GPe activation may offer a new therapeutic strategy for the restoration of PD motor symptoms. Normalization of GPe inhibitory control over the STN might also have a long-term beneficial effect on disease progression, as overactivity and excitotoxicity of glutamatergic projections from the STN to the SN have been implicated in dopaminergic neurotoxicity and the progression of the disease [69,70]. Here, we explored the potential therapeutic effect of global GPe photostimulation, which impacts the function of all neurons. Given the functional heterogeneity of GPe output neurons, the use of a more refined neuromodulation strategy may be required. Further preclinical studies selectively stimulating the different neuronal subpopulations are therefore needed to better characterize the utility of GPe as a target for treating PD.

## 4. Materials and Methods

### 4.1. Subjects

Eight-week-old C57BL/6J (BL6) male mice were purchased from Charles River Laboratories (Saint Germain Nuelles, France). Mice were housed in groups of 3–4 in individually ventilated cages (Techniplast, Grostenquin, France) and kept in 12 h light/dark cycle (light on at 07:00 am, off at 07:00 pm) with water and food ad libitum. All experimental procedures were approved by the French national ethics committee (CE071) and conducted in accordance with EEC (2010/63/UE) guidelines for the care and use of laboratory animals.

### 4.2. Viral Constructions

For global optical stimulation of GPe neurons, the excitatory channelrhodopsin 2 variant (ChR2(H134)) fused to a yellow fluorescent protein (eYFP) was expressed under the hSYN1 promoter using an adeno-associated virus serotype 2 vector (AAV2-hSYN1-ChR2(H134R)-eYFP, 5.6 × 10^12^ particles/mL, Table 1). For optical inhibition, we used the AAV2 vector encoding the engineered chloride channel, IC++, fused to eYFP under the hSYN1 promoter (AAV2-hSYN1-IC++-eYFP, 4.1 × 10^12^ particles/mL). AAV2 encoding the eYFP reporter protein only (AAV2-hSyn1-eYFP, 2 × 10^12^ particles/mL) was used as a control. All viral vectors were obtained from Vector Core (University of North Carolina, Chapel Hill, NC, USA, and MTA from Karl Deisseroth at Stanford University).

### 4.3. Stereotaxic Surgery

#### 4.3.1. Unilateral 6-OHDA Lesion

Mice were pre-treated subcutaneously with buprenorphine (0.05 mg/kg) for analgesia, then deeply anesthetized with isoflurane (4% induction then 1–2%) and placed in a stereotaxic frame (David Kopf instruments, Tujunga, CA, USA) on a headed pad and OcryGel was used to protect from dry eyes. Lidocaine (6 mg/kg) was injected subcutaneously before incising the scalp. Mice received 1.5 µL infusion of 6-OHDA hydrobromide (2.7 µg/µL, free base, Tocris, Bristol, UK, Table 1) either into the left or right SNc over 5 min at the following coordinates: AP: 3 mm, ML ± 1.3 mm and DV −4.7 mm, according to the atlas of Paxinos and Watson, (2001). The injection site was randomized between experimental groups. The microinjector was slowly removed 5 min after the end of the injection to minimize backflow. Control mice (Sham) received an injection of the corresponding volume of vehicle (0.02% ascorbic acid in NaCl) in the same conditions.

#### 4.3.2. Viral Infusion and Fiber Optic Implantation

This procedure was performed either immediately after 6-OHDA lesion for studying the effects of GPe optostimulation in PD mice or in separate surgery session in normal mice for assessing behavioral effects of optogenetic manipulations of GPe activity in a baseline condition. For GPe photostimulation in PD mice or in non-lesioned control mice (Sham), 300 nl of AAV2-hSyn1-ChR2(H134R)-eYFP or AAV2-hSyn1-eYFP (Table 1) was unilaterally infused into the GPe over 6 min (AP: −0.3 mm, ML: ± 2.0 mm and DV: −3.8 mm from the skull surface, according to the atlas of Paxinos and Watson, 2001). The microinjector was slowly removed 6 min after the end of the injection. Following viral injection, an optical fiber-ferrule (optical fiber 200 µm diameter, 0.22 N.A., Doric lenses, Quebec, QC, Canada) was unilaterally implanted 0.2 mm above the injection site and cemented to the skull with Super-Bond C&B. For GPe photoinhibition in a normal condition, normal mice received bilateral infusion of 300 nl of AAV2-hSyn1-iC++-eYFP or AAV2-hSyn1-eYFP in GPe over 6 min at the same stereotaxic coordinates as above. Optical fiber-ferrules were bilaterally implanted 0.2 mm above the injection site and cemented to the skull with Super-Bond C&B.

Animals received a subcutaneous injection of carprofen (3 mg/kg, diluted in NaCl 0.9% and glucose 5%) immediately after surgery and then for two consecutive postoperative days. They were kept for 2–3 weeks in the post-operative room and their weight was monitored daily. Lesioned mice received supplemental food (diet gel, Nutella, chocolate puffed cereals) and additional NaCl 0.9% injections if needed until complete recovery.

### 4.4. Optogenetic Manipulations

Mice were connected through an optical patch cable (Doric lenses, Quebec, QC, Canada) coupled either to a green DPSS laser (532 nm, LaserGlow Technologies, Toronto, CN, Canada) or a blue LD Fiber Light Source (450 nm, LDFS, Doric Lenses, Quebec, QC, Canada) via a rotary joint (Doric Lenses, Quebec, QC, Canada). The light pulses were triggered by programmable TTL Pulse Train Generators (custom-made). The optical light power was calibrated before behavioral testing by measuring the output power at the tip of an optical fiber-ferrule using a light power meter (PM100D, Thorlabs, Le Mesnil-le-Roi, France).

### 4.5. Behavioral Procedures

Behavioral testing was carried 5–6 weeks after the surgery during the light cycle between 09:00 am and 05:00 pm. All experiments were performed on 20–22 weeks-old mice (30 and 28 g mean weight for controls and lesioned mice, respectively). Mice were handled and habituated to the optical connection a few days before behavioral testing. Pilot tests were carried out in a viewing jar (20 cm diameter and 30 cm height Plexiglas cylinder) to establish adequate optical stimulation parameters (light pulse frequency, duration, and intensity) before behavioral testing [71]. For optogenetic studies in 6-OHDA-lesioned mice, a green wavelength light (DPSS laser) was used for photostimulation because the delivery of the blue LDFS was delayed due to the COVID-19 pandemic, and pilot tests carried in the viewing jar showed that it is effective in normal mice (Supplementary Figure S3 for behavioral comparison conducted afterward with green DPSS laser and blue LDFS, see also [57]). For the GPe photostimulation study, mice were first submitted to the open field test for assessing circling behavior and changes in spontaneous locomotor activity, then the cylinder test for assessing forelimb use impairment and postural asymmetry. For the GPe photoinhibition study, mice were tested in the following order: open field test, then the nose-poke habituation test session 1, Y-maze test, nose-poke habituation test session 2, and the cylinder test.

#### 4.5.1. Cylinder Test

Mice were placed in a small Plexiglas cylinder (10 cm diameter, 20 cm height) and forepaw contacts performed against the wall during vertical exploration (rearing) were manually scored using an ethological keyboard (LabWatcher, ViewPoint, Lyon, France) [72]. For studying the effects of GPe optostimulation in PD mice testing was conducted as follows: after 30 sec of familiarization, mice received light pulses for 1 min (ON period) followed by no stimulation for 1 min (OFF period), repeated three times (6 min testing). For GPe photoinhibition in normal mice, the illumination was applied during the whole testing session (6 min). For both studies, testing was conducted only once, and all animals were naïve to the cylinder test. Mice failing to reach three contacts during the 3 min OFF or ON period were excluded from the analyses. One mouse from the 6-OHDA/ChR2 group and one from the iC++ group were excluded. Data are expressed as a percentage of contralateral forepaw contacts relative to the total number of contacts.

#### 4.5.2. Open Field Test

Testing was carried out in a white PVC arena (50 × 50 × 45 cm) and spontaneous locomotor activity was recorded by a video-tracking system (ViewPoint, Paris, France) [73]. Each mouse was individually placed facing a corner of the arena and allowed to freely explore the apparatus. For GPe photostimulation in PD mice, testing was conducted only once, and all animals were naïve to the open field arena. Light pulses were delivered as follows: 2 min OFF periods (baseline locomotor activity level) and 2 min ON periods, repeated twice (8 min testing in total) to assess whether the behavioral effect of the photostimulation was reversible. For GPe photoinhibition in normal mice, testing was repeated to evaluate the behavioral effects of various illumination parameters. We first used a photoinhibition protocol with a repeated 2 min OFF/ON illumination as before. A continuous illumination protocol of 10 min was then used. In each period, distance traveled, velocity, and percentage of time spent in immobility (movement threshold <1 cm/s) were analyzed. Circling behaviors were hand-scored in parallel.

#### 4.5.3. Y-Maze Test

Testing was conducted in mice naïve to the task. Animals were placed in a Y-maze made of grey Plexiglas with three identical arms (40 × 9 × 16 cm) placed at 120° from each other. Each arm had walls with specific motifs allowing it to distinguish one from the others. Mice freely explored the maze for 6 min with the light ON. Spontaneous alternation performance was assessed by visually recording for each mouse the pattern of entrance into each arm in the maze [71]. Alternations were operationally defined as successive entries into each of the three arms as on overlapping triplet sets. Percent spontaneous alternation performance was defined as the ratio of actual (total alternations) to possible alternations (total arm entries -2) × 100. Total entries were scored as an index of exploratory activity in the maze. The latency to exit the starting arm was used to assess neophobia.

#### 4.5.4. Nose-Poke Habituation Test

Testing was conducted in mice naïve to the task. Animals were placed in five-choice operant chambers (Coulbourn Instruments, Allentown, PA, USA) dimly lit with a permanent house light [46]. The front panel was curved and composed of five bays filled with metal wall panels interchangeable by nose-poke modules (Model H21–10 M). Each nose-poke hole was equipped with a controlled yellow LED cue light at the end and infrared photobeam across the opening that detects the nose-pokes. Two adjacent nose-poke units (spaced 4 cm apart) were presented in the right corner and two others in the left corner of the front wall (no nose-poke unit in the central bay). The back wall was composed of a single bay fitted with metal panels and the plexiglass side walls was completely covered by cardboard with distinguishable geometrical motifs. The stainless-steel rod floor (the grid shock floor provided by the manufacturer) was covered by a grey vinyl-coated paper that was used as the standard flooring throughout the study. Testing consisted of 15 min long sessions with nose-poke holes illuminated. The bilateral photoinhibition (continuous illumination) was applied only the first day. One week retention delay was used to assess the impact of GPe inhibition on long-term habituation performances of mice. The number of nose poking was monitored and used as an index of directed exploration and habituation capacity to salient spatial cues.

### 4.6. Immunofluorescence and Histological Analysis

#### 4.6.1. Analysis of 6-OHDA-Induced Dopamine Cell Loss

Mice were perfused transcardially with 4% paraformaldehyde and the brains were removed, postfixed, and cryoprotected. Free-floating coronal sections (40 µm thick) containing the SNc were incubated with rabbit anti-TH antibody (1/1000, Synaptic Systems, 213 102, Göttingen, Germany) overnight at 4 °C followed by a 2 h incubation with the Alexa Fluor 594 goat anti-rabbit antibody (1/400, Jackson Laboratory, Bar Harbor, MA, USA, 111-585-003) at room temperature (RT). Sections were mounted onto SuperFrost Plus glass slides (VWR) and coverslipped with Roti^®^-Mount FluorCare mounting medium (Carl Roth). Immunofluorescence was analyzed by laser confocal microscopy (Zeiss LM710, Jena, Germany) at a 20 × magnification. TH positive cells were counted through the SNc region based on the stereotaxic mouse atlas (Paxinos & Franklin, 2001) delineating the SNc from the ventral tegmental area by the medial optic tract. Counting was done manually in five regions of interest (ROIs) per hemisphere, covering the whole mediolateral extension of the structure (−2.70 to −3.80 mm relative to bregma, three sections per mouse), using cell counter plugin of FIJI software (ImageJ, National Institutes of Health, Bethesda, MD, USA).

DA depletion in the striatum was also examined. Coronal sections (40µm) were incubated overnight at 4 °C with a mouse anti-TH antibody (1/1000, Millipore, MAB318). Thereafter, they were incubated with a biotinylated secondary antibody (goat anti-mouse, 1/200; Jackson Immunoresearch; 115-065-003) for 1 h and then in a solution containing 0.01% DAB (3,3-diaminobenzidine) and 3% H_2_O_2_ diluted in PBS 1× for 3 min. Sections were finally mounted on slides as described before. Images were acquired using a bright field microscope (Nikon Leica DMLB) at 10× magnification.

#### 4.6.2. Analysis of Opsins Expression

Free-floating coronal sections (40 µm thick) containing the GPe were incubated a 48 h with a rabbit anti-GFP antibody (1/500, Invitrogen, A-11122, Waltham, MA, USA) at 4 °C then 1 h 30 with Alexa Fluor 488 donkey anti-rabbit antibody (1/500, Invitrogen, R37118, Waltham, MA, USA) at RT. Sections were mounted onto SuperFrost Plus glass slides (VWR) and coverslipped with Roti^®^-Mount FluorCare mounting medium (Carl Roth). Immunofluorescence was analyzed by laser confocal microscopy (Zeiss LM710, Jena, Germany) at 10× magnification.

#### 4.6.3. Verification of Optic Fiber-Ferrule Placement

For confirming the placement of the optical fiber in the GPe, coronal sections of the GPe were stained with cresyl violet. Images were acquired using Leica DM LB Microscope at 2.5× magnification equipped with Nikon digital camera DXM1200. Mice with optical fiber-ferrule placements outside of the GPe were excluded from the analysis.

### 4.7. Statistical Analysis

All data are expressed as mean group value ± standard error of the mean (s.e.m.). All statistical analyses were performed with GraphPad Prism software version 9 (GraphPad Software, Boston, MA, USA). Shapiro–Wilk test was first used to ensure that the assumption of normality was not violated. Based on the results, the non-parametric Kruskall–Wallis test followed by Dunn’s post hoc test was used for the analysis of circling behavior, forelimb akinesia, and rears data of the study on 6-OHDA-lesioned mice. The non-parametric Mann–Whitney test was used for the analysis of circling behavior, forelimb akinesia, and rears data of the study on iC++ mice. For all studies, locomotor activity data in the open field test were analyzed using two-way ANOVA with repeated measures (OFF and ON periods or time). When relevant, post hoc comparisons were carried out using a *t*-test or Tukey’s multiple comparisons tests. The accepted level of significance was *p* < 0.05.

## Figures and Tables

**Table 1:** Key resources.

REAGENT	SOURCE	IDENTIFIER
**Chemicals**		
Cresyl violet	Sigma Aldrich, saint-quentin-fallavier, France	C5042
Buprenorphine (Vetergesic^®^)	Centravet, Maisons-Alfort, France	VET063
Carprofene (Rimadyl^®^)	Centravet, Maisons-Alfort, France	RIM011
Pentobarbital (Euthasol^®^)	Centravet, Maisons-Alfort, France	EUT002
Paraformaldehyde	Carl Roth, Karlsruhe, Germany	4980.2
Isoflurane	Virbac, Carros, France	200265
**Vectors**		
AAV2-hSYN1-ChR2(H134R)-eYFP	UNC Vector Core, Chapel Hill, NC, USA	N/A
AAV2-hSYN1-IC++-eYFP	UNC Vector Core, Chapel Hill, NC, USA	N/A
AAV2-hSyn1-eYFP	UNC Vector Core, Chapel Hill, NC, USA	N/A
**Antibodies**		
Rabbit anti-GFP	Invitrogen, Massy, France	A-11122
Rabbit anti-TH	Synaptic Systems (SYSY), Göttingen, Germany	213102
Mouse anti-TH	Millipore, Darmstadt, Germany	MAB318
Alexa Fluor 488 donkey anti-rabbit	Invitrogen, Massy, France	R37118
Alexa Fluor 594 goat anti-rabbit	Jackson ImmunoResearch, Ely, UK	111-585-003
Biotin goat anti-mouse	Jackson ImmunoResearch, Ely, UK	115-065-003

## Data Availability

The data reported in this paper will be shared by the lead contact upon reasonable request.

## Supplementary Materials

The supporting information can be downloaded at: https://www.mdpi.com/article/10.3390/ijms24097935/s1.
