# Supplementary material for: Optogenetic Globus Pallidus Stimulation Improves Motor Deficits in 6-Hydroxydopamine-Lesioned Mouse Model of Parkinson’s Disease
Source: Int J Mol Sci. 2023 Apr 27;24(9):7935. doi: 10.3390/ijms24097935 (PMC10178372; doi:10.3390/ijms24097935)
Supplement: Supplementary file 1 [file ijms-24-07935-s001.zip › ijms-2222642-supplementary.pdf]

International Journal of
Molecular Sciences

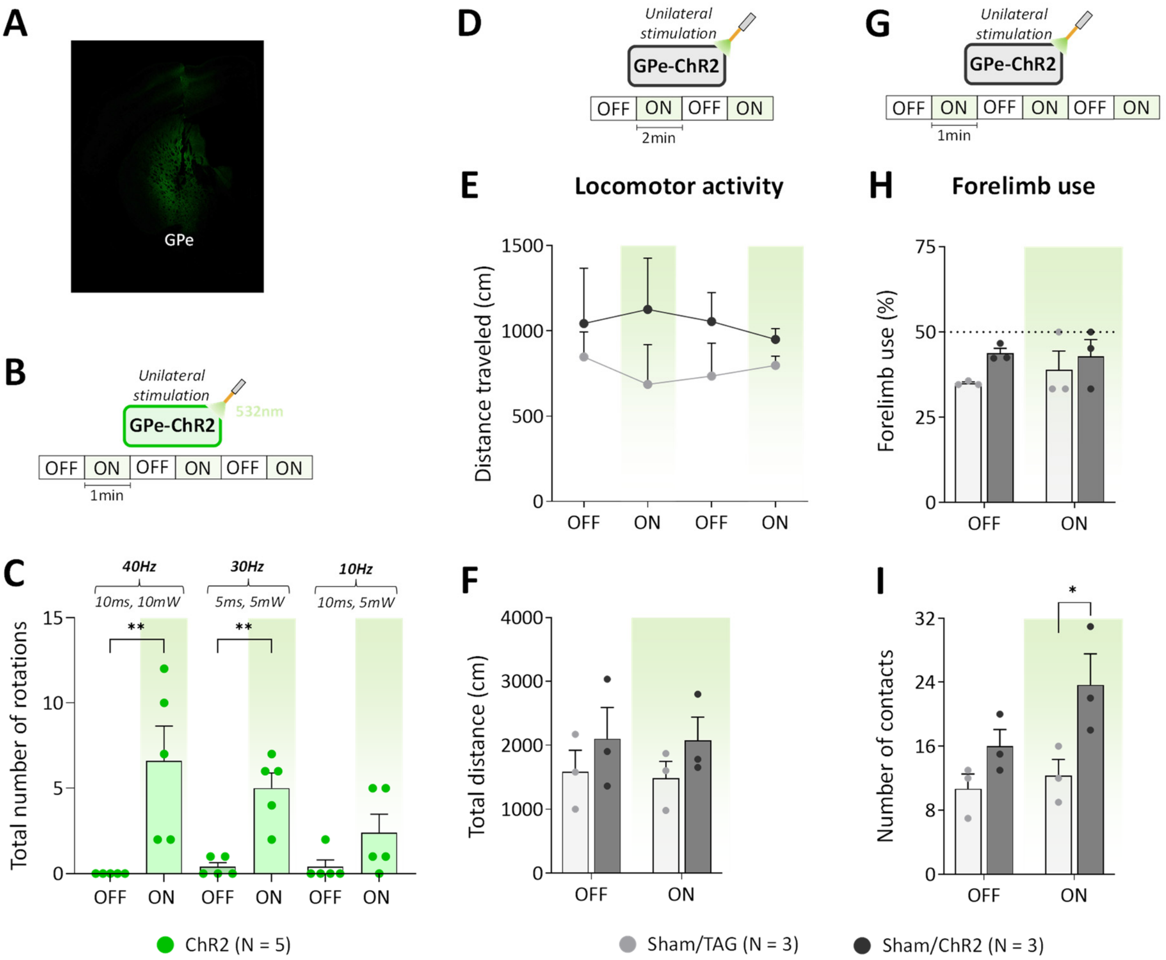

Supplementary Figure S1: Behavioral effect of global GPe photostimulation in Sham controls group. (A) Immunofluoresence image illustrating unilateral eYFP expression in GPe neurons. (B) Photostimulation protocol for the viewing jar test. Testing was carried over 6 min with repeated sequences of light stimulation turned OFF and ON for 1 min. (C) Total number of contralateral rotations during OFF and ON periods for each stimulation tested. (D) Photostimulation protocol used for the open field test. Testing was carried over 8 min with repeated sequences of light stimulation (532 nm, 5 Hz, 5 ms, 3 mW) turned OFF and ON for 2 min. (E-F) Time course and total distance traveled (cm) during OFF and ON periods, respectively. (G) Photostimulation protocol used for the cylinder test. Testing was carried over 6 min with repeated sequences of light stimulation (532 nm, 5 Hz, 5 ms, 3 mW) turned OFF and ON for 1 min. (H) Percentage of contralateral forelimb use relative to the total number of forepaw contacts during OFF and ON periods. (I) Total number of rears (forepaw contacts) during OFF and ON periods. ChR2, N = 5; Sham/TAG, N = 3; Sham/ChR2, N = 3. Results are presented as mean ± SEM. *$p<0.05$, Mann-Whitney test.

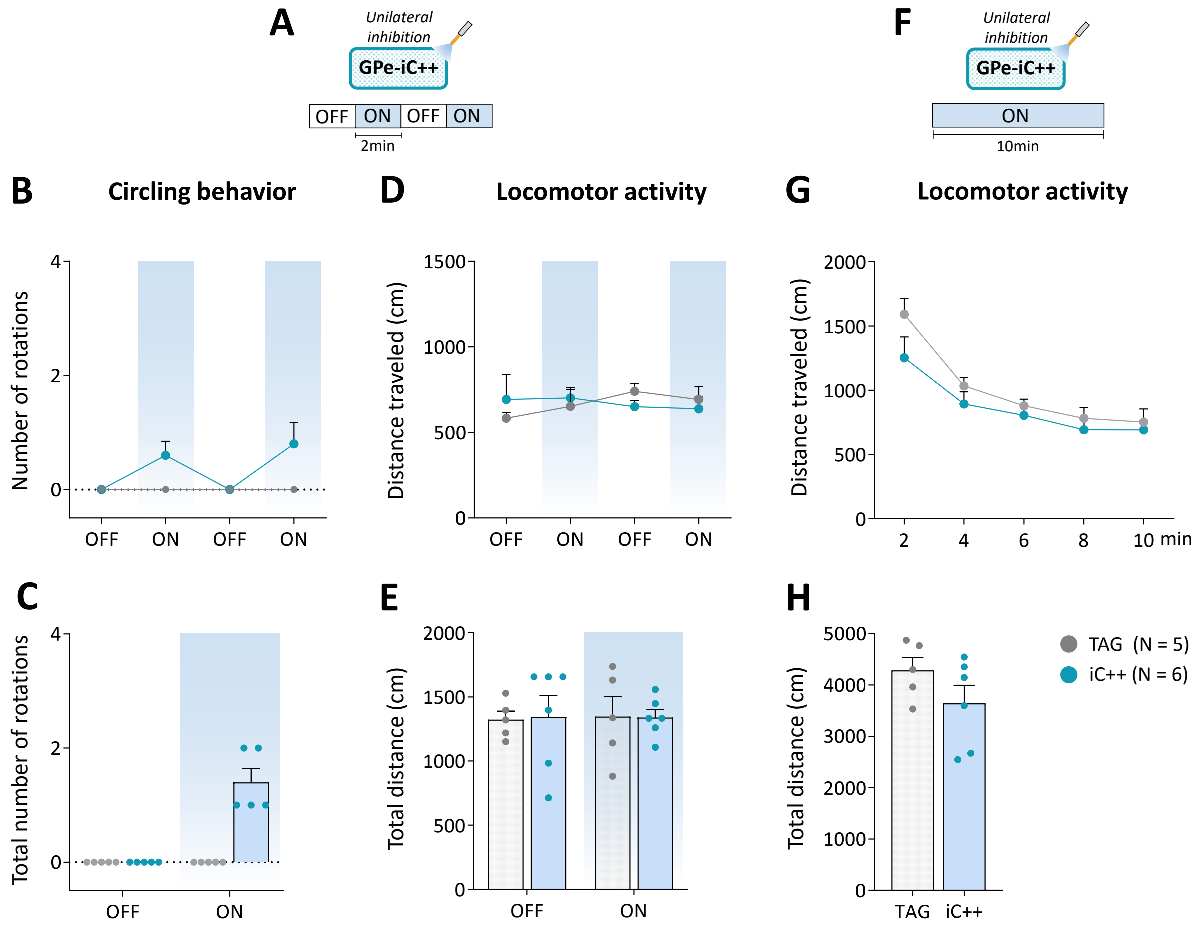

Supplementary Figure S2. Global GPe photoinhibition has no impact on locomotor behavior in normal mice. (A) Photoinhibition protocol for the open field test. Testing was carried over 8 min with repeated sequences of unilateral light pulses stimulation (450 nm, 60 Hz, 10 ms, 12 mW) turned OFF and ON for 2 min. (B-C) Time course and total number of contralateral rotations during OFF and ON periods, respectively. (D-E) Time course and total distance traveled (cm) during OFF and ON periods, respectively. (F) Photoinhibition protocol used for the open field test. Testing was carried over 10 min with continuous light illumination (450 nm, 12 mW). (G-H) Time course and total distance traveled (cm) over 10 min of testing, respectively. TAG, N = 5; iC++, N = 6. Results are presented as mean ± SEM.

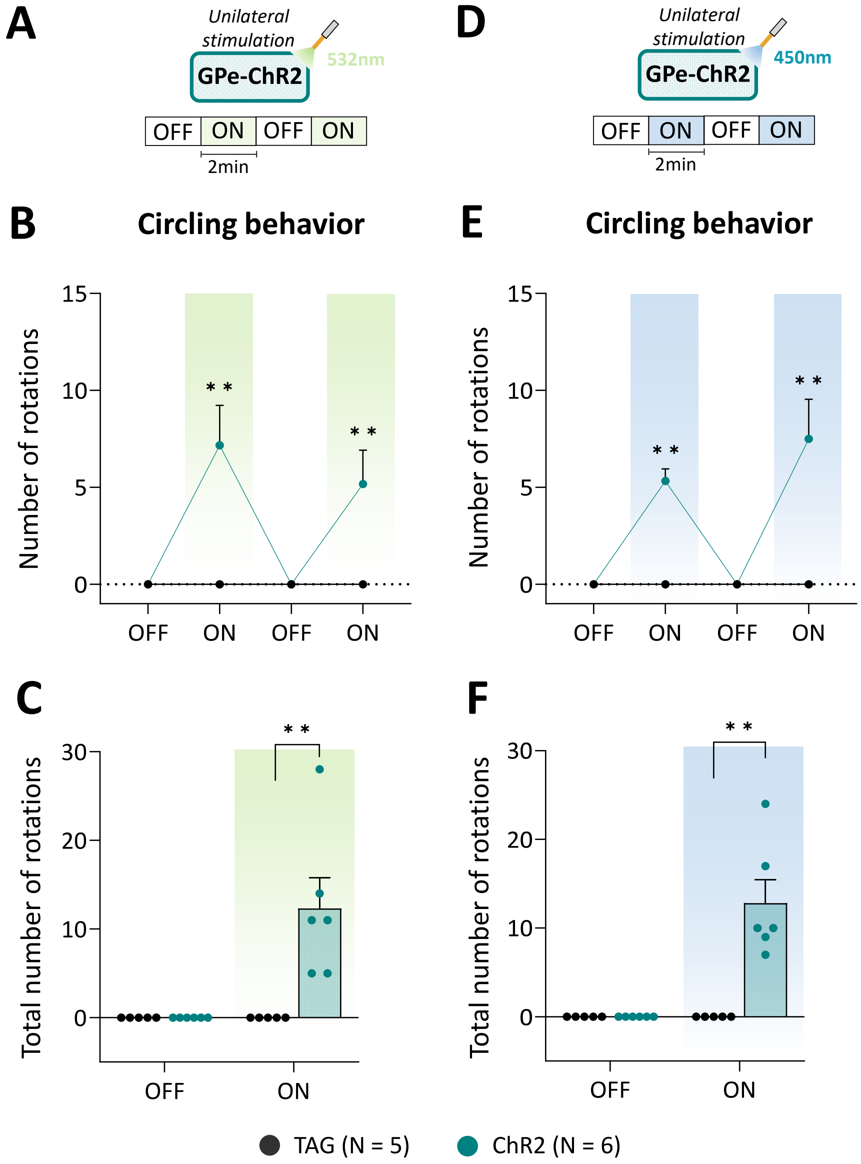

Supplementary Figure S3. Similar behavioral effects of GPe photostimulation with green or blue light in normal mice. (A) Photostimulation protocol for the open field test. Testing was carried over 8 min with repeated sequences of light stimulation (532 nm, 30 Hz, 5 ms, 3 mW) turned OFF and ON for 2 min. (B-C) Time course and total number of contralateral rotations during OFF and ON periods, respectively. (D) Photostimulation protocol for the open field test. Testing was carried over 8 min with repeated sequences of light stimulation (450 nm, 30 Hz, 5 ms, 3 mW) turned OFF and ON for 2 min. (E-F) Time course and total number of contralateral rotations during OFF and ON periods, respectively. TAG, N = 5; iC++, N = 6. Results are presented as mean ± SEM. **p < 0.01, Mann-Whitney test.
